# Grape Seed Proanthocyanidins Inhibit Replication of the Dengue Virus by Targeting NF-kB and MAPK-Mediated Cyclooxygenase-2 Expression

**DOI:** 10.3390/v15040884

**Published:** 2023-03-30

**Authors:** Wei-Chun Chen, Monir Hossen, Wangta Liu, Chia-Hung Yen, Chung-Hao Huang, Yao-Chin Hsu, Jin-Ching Lee

**Affiliations:** 1 Department of Marine Biotechnology and Resources, College of Marine Sciences, National Sun Yat-sen University, Kaohsiung 80424, Taiwan; daphny3016@hotmail.com; 2 Department of Biotechnology, College of Life Science, Kaohsiung Medical University, Kaohsiung 80708, Taiwan; liuwangta@cc.kmu.edu.tw; 3 Graduate Institute of Medicine, College of Medicine, Kaohsiung Medical University, Kaohsiung 80708, Taiwan; monir.mbg@gmail.com; 4 Graduate Institute of Natural Products, College of Pharmacy, Kaohsiung Medical University, Kaohsiung 80708, Taiwan; chyen@kmu.edu.tw; 5 Division of Infectious Diseases, Department of Internal Medicine, Kaohsiung Medical University Hospital, Kaohsiung Medical University, Kaohsiung 80708, Taiwan; 960277@mail.kmuh.org.tw; 6 School of Medicine, Graduate Institute of Medicine, College of Medicine, Sepsis Research Center, Kaohsiung Medical University, Kaohsiung 80708, Taiwan; 7 Center for Tropical Medicine and Infectious Disease, Kaohsiung Medical University, Kaohsiung 80708, Taiwan; 8 Department of Chinese Medicine, Chi Mei Medical Center, Tainan 71004, Taiwan

**Keywords:** dengue virus, grape seed extract, cyclooxygenase, inflammation

## Abstract

Dengue virus (DENV) infection is a serious global health issue as it causes severe dengue hemorrhagic fever and dengue shock syndrome. Since no approved therapies are available to treat DENV infection, it is necessary to develop new agents or supplements that can do this. In this study, grape seed proanthocyanidins extract (GSPE), which is widely consumed as a dietary supplement, dose-dependently suppressed the replication of four DENV serotypes. The inhibitory mechanism demonstrated that GSPE downregulated DENV-induced aberrant cyclooxygenase-2 (COX-2) expression, revealing that the inhibitory effect of the GSPE on DENV replication involved targeting DENV-induced COX-2 expression. Mechanistic studies on signaling regulation have demonstrated that GSPE significantly reduced COX-2 expression by inactivating NF-κB and ERK/P38 MAPK signaling activities. Administrating GSPE to DENV-infected suckling mice reduced virus replication, mortality, and monocyte infiltration of the brain. In addition, GSPE substantially reduced the expression of DENV-induced inflammatory cytokines associated with severe dengue disease, including tumor necrosis factor-α, nitric oxide synthase, interleukin (IL)-1, IL-6, and IL-8, suggesting that GSPE has potential as a dietary supplement to attenuate DENV infection and severe dengue.

## 1. Introduction

Dengue fever caused by viral (DENV) infection is the most widespread mosquito-borne infectious viral disease in tropical and subtropical regions [1]. DENV is a positive-sense single-stranded RNA-enveloped virus with four serotypes (DENV-1–DENV-4) which have 65–70% nucleotide sequence homology. The virus is part of the *Flavivirus* genus (*Flaviviridae* family). The DENV genome is approximately 11 kb long and encodes three structural proteins; including the envelope, capsid, and pre-membrane proteins; and seven non-structural proteins; including NS1, NS2A, NS2B, NS3, NS4A, NS4B, and NS5 [2]. According to the World Health Organization (WHO), about 500,000 individuals are diagnosed with severe DENV infection each year, of which about 2.5% die from the disease [3]. The clinical manifestations of DENV infection vary from asymptomatic or symptomatic dengue fever to severe dengue hemorrhagic fever (DHF) and dengue shock syndrome (DSS) [4]. Subsequent infection with a different serotype is known as a secondary DENV infection and is associated with a high risk of death from severe DHF or DSS [5]. The hallmark pathogenic features of DHF are vascular endothelial dysfunction and leakage, which can progress to more fatal syndromes such as hemorrhage, coagulopathy, and thrombocytopenia [6]. The reason for the vascular leakage in DHF patients is the massive release of pro-inflammatory cytokines and chemokines upon viral infection, such as interleukin-6 (IL-6), IL-8, IL-1β, vascular endothelial growth factor, and tumor necrosis factor-α (TNF-α), leading to endothelial cell damage [7]. While DENV infection is an alarming global issue, there is currently no effective antiviral treatment or vaccine. Therefore, discovering and developing new therapeutics or supplements for dengue treatment is an urgent matter.

The key rate-limiting enzyme in the conversion of arachidonic acid to prostaglandins is prostaglandin-endoperoxide synthase, also known as cyclooxygenase (COX). COX-1 and COX-2 are the two isoforms, which are considered constitutive and inducible enzymes, respectively. A previous study revealed that DENV infection stimulates the pro-inflammatory mediator COX-2 to be overexpressed via the NF-kB and mitogen-activated protein kinase (MAPK) signaling pathways, which facilitates DENV replication that results in pathogenesis. In contrast, silencing COX-2 expression and its activity using a COX-2 shRNA or selective COX-2 inhibitor inhibits DENV replication [8]. Several natural products effectively suppress viral-induced COX-2 expression, revealing their potential as supplements for DENV-related pathogenesis [9,10,11]. Thus, downregulating DENV-mediated COX-2 expression with inhibitors is a promising strategy to suppress DENV replication as well as DENV-induced inflammatory pathogenesis. 

Grape seed extract includes many phenolic compounds, such as (+) catechin, gallic acids, epicatechin, dimeric procyanidin, and proanthocyanidins, which are promising bioactive elements for many diseases [12]. Grape seed proanthocyanidins extract (GSPE) is a widely used dietary supplement that exhibits an anti-inflammatory profile [13,14,15,16] along with antioxidant properties [17,18], antimicrobial effects [19,20], and antiviral effects [21,22,23]. We previously revealed that a GSPE treatment significantly suppresses hepatitis C virus (HCV)-elevated COX-2 expression as well as HCV replication [24]. Here, we examined the anti-DENV activity of GSPE in vitro and in vivo and performed mechanistic studies of the effects of GSPE on DENV replication. Additionally, anti-DENV-induced inflammation was examined. The findings support the use of GSPE as an alternative supplement to control DENV infection and DENV-associated diseases.

## 2. Materials and Methods

### 2.1. Ethics Statement

The ICR strain mice were purchased from BioLASCO Taiwan Co., Ltd. (Taipei, Taiwan). All animal experiments were performed according to the *Guide for the Care and Use of Laboratory Animals*. The experimental procedures were approved by the Animal Research Committee of Kaohsiung Medical University of Taiwan (IACUC # 109184) under the guidance of the Public Health Service Policy on Human Care and Use of Laboratory Animals. 

### 2.2. Cell Culture and Virus 

Huh-7 hepatoma cells were maintained in Dulbecco’s modified Eagle’s medium with 10% heat-inactivated fetal bovine serum (FBS), 1% antibiotic-antimycotic, and 1% non-essential amino acids. The cells were incubated at 37 °C with a 5% CO_2_ supplement. DENV type-1 (DENV-1: DN8700828), type-2 (DENV-2: 16681), type-3 (DENV-3: DN8700829A), and type-4 (DENV-4: S9201818) were obtained from the Center for Disease Control (CDC), Department of Health, Taiwan. All serotypes were amplified in the C6/36 cell line according to the procedure described in a previous study [25].

### 2.3. Reagents

The IH636 GSPE was purchased from InterHealth Nutraceuticals (Benicia, CA, USA) [26]. The GSPE was dissolved in DMSO to a final concentration of 0.1% and diluted in DMEM medium.

### 2.4. A Cell-Based Anti-DENV Activity Assay

Huh-7 cells were seeded in 24-well plates (5 × 10^4^ /well) and then infected with DENV2 at an MOI of 0.1 for 2 h. After removing the inoculum, the DENV-infected Huh-7 cells were treated with various concentrations of GSPE for 3 days. The DENV protein and RNA levels were then analyzed by western blotting and RT-qPCR, respectively. The relative DENV RNA levels were presented as percent changes compared to the GSPE-untreated cells, which represented 100%. The relative DENV protein levels were presented as fold-change values compared to the GSPE-untreated cells. The value of GSPE-untreated cells was found to be 1 and was determined by quantifying the band intensity in the X-ray film of a western blot in which cellular GAPDH served as a loading control for normalization of band intensity using the ImageJ software (National Institutes of Health, Bethesda, MD, USA).

### 2.5. Western Blotting Assay

Western blotting was carried out as described previously [27]. In brief, an equal volume of cellular lysate was analyzed by sodium dodecyl sulfate-polyacrylamide gel electrophoresis, and the proteins were transferred to a PVDF membrane. The membranes were probed with antibodies specific to DENV NS2B (1:5000; Abcam, Cambridge, MA, USA), anti-GAPDH (1:10,000; GeneTex, Irvine, CA, USA), anti-COX-2 antibody (1:1000; Cayman Chemical, Ann Arbor, MI, USA), anti-MAPK (phosphorylated and unphosphorylated forms of ERK1/2, p38, and JNK), and anti-C-Myc antibody (1:1000; GeneTex). 

### 2.6. Real-Time Quantitative Reverse Transcription-Polymerase Chain Reaction (RT-qPCR)

Total RNA was extracted with an RNA extraction kit (GMbiolab, Co., Ltd., Taichung City, Taiwan) according to the manufacturer’s instructions. The levels of DENV NS5, TNF-α, IL-1β, inducible nitric oxide synthase (iNOS), and COX-2 RNA were quantified by RT-qPCR as described previously [25], using the primers listed in Table 1. The relative RNA levels of the genes in each sample were normalized using cellular *gapdh* mRNA.

### 2.7. Cytotoxicity Assay 

Cell viability was assessed according to the procedure described in our previous study [24]. In brief, Huh-7 cells (5 × 10^3^ cells/well in a 24-well plate) were exposed to various concentrations of GSPE. After 3 days of incubation, relative cell viability was assessed using the CellTiter 96^®^ AQueous One Solution Cell Proliferation Assay (MTS, Promega Corp., Madison, WI, USA) according to the manufacturer’s instructions. Color intensity was detected at 490 nm on a microplate reader (BioTek, Norcross, GA, USA).

### 2.8. Transfection and Luciferase Activity Assay 

pCOX-2-Luc containing the COX-2 promoter region driving firefly luciferase was used to measure COX-2 transcriptional activity. pNF-κB-Luc containing NF-κB binding element-driven firefly luciferase was used to measure NF-κB transcriptional activity. pCMV-COX-2-Myc encoding the COX-2 gene was used to determine exogenous COX-2 expression [8]. Huh-7 cells (5 × 10^4^ cells/well in a 24-well plate) were transfected for 8 h with the desired plasmid using the T-Pro^TM^ reagent (Ji-Feng Biotechnology, Co., Ltd., Taipei, Taiwan) according to the manufacturer’s instructions. After transfection, the medium was replaced with various concentrations of GSPE for 3 days. The luciferase assay was performed using the Bright-Glo^TM^ Luciferase Assay System (Promega, Madison, WI, USA) according to the manufacturer’s protocol.

### 2.9. Preparation of the Nuclear Fraction

Huh-7 cells (4 × 10^5^ cells/well in a 24-well plate) were treated with or without GSPE at the indicated dose. After a 3-day-long incubation, the cells were lysed and the nuclear extracts were prepared using hypotonic [10 mM HEPES, 1.5 mM MgCl_2_, 10 mM KCl, 0.5 mM DTT, 10% Nonidet P-40 (pH 7.9)] and high-salt buffer [20 mM HEPES, 1.5 mM MgCl_2_, 0.2 mM EDTA, 0.6 M KCl, 0.5 mM DTT (pH 7.9)] solutions, as described previously [28]. 

### 2.10. In Vivo Anti-DENV Activity Assay 

Six-day-old ICR suckling mice were randomly divided into three groups (five mice/group). The mice were intracerebrally injected with a virus and GSPE. Group 1 received 60 °C heat-inactivated DENV-2 and the saline treatment (iDENV), group 2 received 1 × 10^5^ plaque-forming units (PFU) of DENV-2 plus the saline treatment (DENV), and group 3 first received 1 × 10^5^ PFU DENV, which was later followed by 20 mg/kg GSPE at 1-, 3-, and 5-days post-infection. The body weights and mortality rates of the mice in each group were recorded daily for up to 6 days. The suckling mice were sacrificed after 6 days by CO_2_ asphyxiation. The brain tissue samples were collected, weighed, and homogenized in 0.5 mL RPMI 1640 medium supplemented with 2% FBS and centrifuged at 8000 rpm for 15 min at 4 °C for the plaque assay.

### 2.11. Plaque Assay 

The plaque assay was assessed according to the procedure described in our previous study [27]. The infectious viral particles from the brain of the DENV-2 infected mice were collected and serially diluted with DMEM. BHK-21 cells were plated in 12-well plates at 1 × 10^5^ cells per well and incubated with the diluted virus in a volume of 400 μL at 30 °C for 2 h. Then, the infected cells were washed with PBS 3 times and 3 mL of DMEM containing 2% FBS and 0.8% methylcellulose (Sigma-Aldrich, St. Louis, MO, USA) was added into each well. After 5 days post-infection, the supernatant was removed and the cells were fixed and stained with the plaque assay solution (1% crystal violet, 0.64% NaCl, and 2% formalin) at 25 °C for 2 h. The viral titer was calculated by observation of plaque formation.

### 2.12. Immunohistochemical (IHC) Assay

IHC staining was performed according to a protocol described previously [29]. The slides were incubated with antibodies against Ly6C (monocyte marker) for the monocyte infiltration analysis. Monocyte infiltration was quantified with a slide scanner (Motic EasyScan Digital Scanner, Hong Kong, China) and ImageJ software (National Institutes of Health, Bethesda, MD, USA).

### 2.13. Statistical Analysis

The results are presented as mean ± standard deviation of at least three independent experiments. The comparisons were analyzed with the Student’s t-test using GraphPad Prism version 8 software (GraphPad Software Inc., La Jolla, CA, USA). A *p*-value < 0.05 was considered significant.

## 3. Results

### 3.1. GSPE Suppresses DENV Replication in Virus-Infected Huh-7 Cells

**Figure 1:**
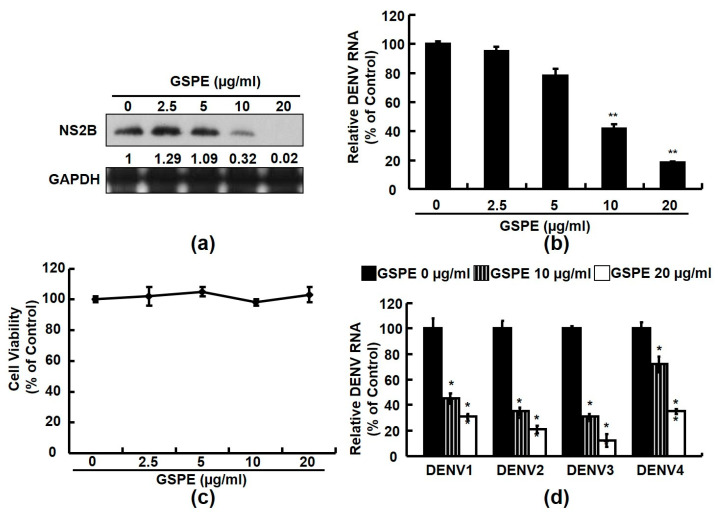
GSPE inhibits DENV protein synthesis and RNA replication. (**a**) DENV protein expression and (**b**) RNA replication decreased in response to the GSPE treatment in DENV-infected Huh-7 cells. Huh-7 cells were seeded in 24-well plates and infected with DENV-2 at an MOI of 0.1 for 2 h. Then, the virus was removed and the DENV-infected Huh-7 cells were treated with GSPE at increasing concentrations (2.5–20 μg/mL) for 3 days. DENV protein synthesis was detected by western blotting and RNA levels were quantified by qRT-PCR. Cellular GAPDH protein served as the equal loading control and mRNA levels served as the internal control for western blotting and RT-qPCR, respectively. The relative DENV NS2B protein levels were presented as fold-change values compared to the GSPE-untreated Huh-7 cells. (**c**) Cytotoxicity was determined by the MTS assay. (**d**) The GSPE treatment inhibited DENV serotypes 1, 2, 3, and 4. Huh-7 cells were seeded in 24-well plates and separately infected with the four DENV serotypes (DENV-1: DN8700828; DENV-2: 16681; DENV-3: DN8700829A; DENV-4: S9201818) at an MOI of 0.1 for 2 h. Then, the DENV-infected Huh-7 cells were treated with the indicated concentrations (2.5–20 μg/mL) of GSPE for 3 days and RNA replication was quantified by RT-qPCR. “0” indicates treatment with 0.1% DMSO. The relative DENV RNA levels and cell viability values are presented as percent changes compared to the GSPE-untreated cells, which represented 100%. Data are presented as mean ± SD of three independent experiments. * *p* < 0.05; ** *p* < 0.01.

Several studies have shown that GSPE has antiviral action against many viruses [21,22,23,24]. To investigate the anti-DENV activity of GSPE, we treated DENV-2-infected Huh-7 cells with increasing concentrations of GSPE (2.5–20 µg/mL). Western blot and RT-qPCR procedures were performed after 3 days to analyze the DENV-2 NS2B protein and viral RNA levels. The results showed that GSPE dose-dependently reduced the DENV protein (Figure 1a) and RNA (Figure 1b) levels, with an EC_50_ value of 10 ± 2 µg/mL. No significant cytotoxicity was detected in the GSPE-treated cells compared to the untreated cells according to the MTS assay (Figure 1c). In addition, we further verified that the GSPE treatment effectively suppressed replication of the four DENV serotypes (Figure 1d).

### 3.2. GSPE Downregulates DENV-Induced COX-2 Expression in Huh-7 Cells

**Figure 2:**
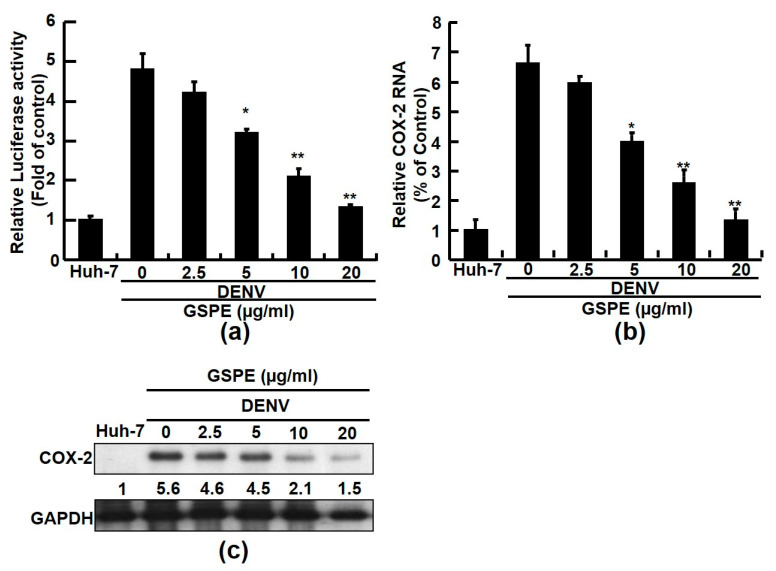
GSPE reduces DENV-induced COX-2 expression. (**a**) The GSPE treatment reduced COX-2 promoter activity in a concentration-dependent manner. Huh-7 cells were transfected with the pCOX-2-Luc reporter plasmid. After 8 h of transfection, the transfected-Huh-7 cells were infected with DENV-2 at an MOI of 0.1 for 2 h and treated with the indicated concentrations (2.5–20 μg/mL) of GSPE for 3 days. The cell lysates were subjected to a luciferase activity assay. Relative COX-2 promoter activity is presented as a fold change compared to parental Huh-7 cells. (**b**,**c**) DENV-induced COX-2 expression decreased in response to GSPE treatment. DENV-infected Huh-7 cells were treated with GSPE at increasing concentrations for 3 days. COX-2 expression was analyzed by western blotting and RT-qPCR, respectively. Cellular GAPDH protein served as an equal loading control and mRNA levels served as an internal control for western blotting and RT-qPCR normalization, respectively. “0” indicates the treatment with 0.1% DMSO. The relative COX-2 RNA and protein levels are presented as fold-change values compared to the Huh-7 cells without viral infection, which had a value of 1. Data are presented as mean ± SD of three independent experiments. * *p* < 0.05; ** *p* < 0.01.

According to our previous study, COX-2-derived prostaglandin E2 (PGE_2_) is associated with modulation of DENV-2 replication and DENV-induced-inflammatory responses [8]. In addition, GSPE was found to attenuate HCV-induced COX-2 expression against viral replication [24]. To investigate whether GSPE also mitigates DENV-induced COX-2 expression, we performed a COX-2 promoter assay to identify the effect of GSPE on DENV-induced transactivation. To accomplish this, the pCOX-2-Luc reporter plasmid was first transfected into Huh-7 cells and then infected with DENV. The transfected cells were then incubated with GSPE at the indicated concentrations for 3 days. The results showed that GSPE gradually decreased the DENV-elevated transactivation of COX-2 in DENV-infected Huh-7 cells (Figure 2a). Next, we conducted RT-qPCR and western blot analyses to examine COX-2 expression in response to the GSPE treatment. Consistent with the promoter-based result, GSPE decreased the DENV-elevated COX-2 RNA and protein levels in a concentration-dependent manner (Figure 2b and c). Taken together, these results suggest that the GSPE treatment significantly reduced DENV-induced COX-2 expression.

### 3.3. GSPE Inhibits DENV Replication by Suppressing COX-2 Expression 

**Figure 3:**
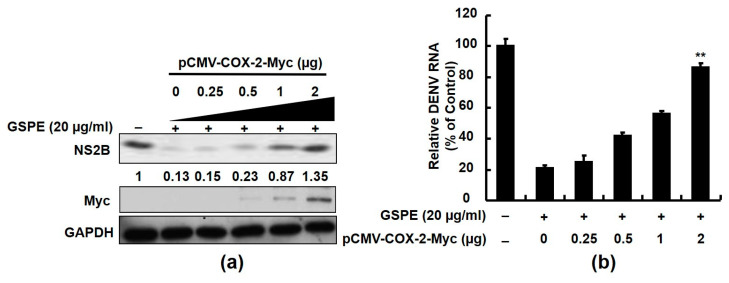
Exogenous overexpression of COX-2 attenuates the anti-DENV activity of GSPE. Huh-7 cells were transfected with the indicated amounts of pCMV-COX-2-Myc (0.25, 0.5, 1, and 2 µg) for 8 h. The transfected cells were infected with DENV-2 at an MOI of 0.1 for 2 h and then treated with 20 μg/mL of the GSPE for 3 days. (**a**) Western blotting was performed with anti-NS2B, anti-Myc, and anti-GAPDH (loading control) antibodies to analyze protein expression. The relative DENV NS2B protein levels were presented as fold-change values compared to the GSPE-untreated Huh-7 cells. (**b**) The DENV RNA levels were quantified by RT-qPCR following normalization of cellular *gapdh* mRNA. Relative DENV RNA levels were presented as percent changes compared to GSPE-untreated/non-transfected Huh-7 cells, which were considered 100%. “0” indicates the transfection of vehicle plasmid. Data are presented as mean ± SD of three independent experiments. ** *p* < 0.01.

Suppressing viral-induced COX-2 expression has been reported to interfere with viral replication [28]. To further clarify whether the attenuation of COX-2 by GSPE was correlated with anti-DENV activity, we transfected Huh-7 cells with increasing concentrations of pCMV-COX-2-Myc, a COX-2 expression vector, and infected them with DENV-2. Then, the cells were treated with 20 µg/mL of GSPE. After 3 days, DENV protein synthesis and RNA replication were measured by western blotting and RT-qPCR, respectively. As shown in Figure 3a, the GSPE-suppressed DENV protein levels were gradually restored by exogenous COX-2-Myc with an increasing amount of transfected plasmid, indicating that COX-2 expression strongly supports DENV replication. Similarly, the DENV RNA levels were also restored in proportion to exogenous COX-2 expression under the GSPE treatment (Figure 3b). Taken together, these findings show that GSPE prevented DENV replication by blocking DENV-elevated COX-2 expression. 

### 3.4. GSPE Modulates the NF-kB and MAPK Pathways Which Inhibit DENV Replication

**Figure 4:**
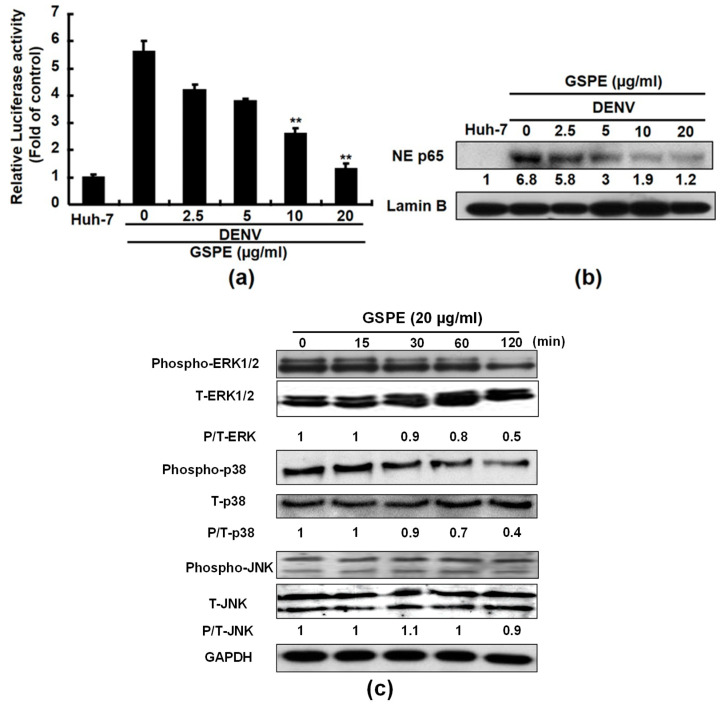
GSPE suppresses COX-2 expression by limiting NF-B transactivity and MAPK phosphorylation in DENV-infected Huh-7 cells. (**a**) GSPE decreased NF-κB transactivity in Huh-7 cells. Huh-7 cells were transfected with the pNF-κB-Luc reporter plasmid. The pNF-κB-Luc-transfected cells were infected with DENV-2 at an MOI of 0.1 for 2 h and then treated with 20 μg/mL of GSPE for 3 days. NF-κB transactivity was analyzed by a luciferase activity assay. Relative NF-κB transactivity is presented as a fold change compared to the parental Huh-7 cells in which luciferase activity was 1. (**b**) The GSPE treatment downregulated nuclear translocation of the p65 NF-κB subunit. DENV-infected Huh-7 cells were treated with different concentrations (2.5–20 μg/mL) of GSPE for 3 days. Nuclear translocation of NF-κB was analyzed by western blotting with anti-phospho-p65 and anti-lamin B (loading control) antibodies. NE indicates the nuclear extract fraction. (**c**) The GSPE treatment reduced the ERK and p38 phosphorylation levels. DENV-infected Huh-7 cells were treated with 20 μg/mL of GSPE and the lysates were extracted at the indicated time points after the treatment. Protein expression was analyzed by western blotting using antibodies against MAPK (ERK1/2, p38, and JNK), phospho-MAPK (p-ERK1/2, p-p38, and p-JNK), and GAPDH (loading control). “0” indicates the treatment with 0.1% DMSO. The relative protein levels were presented as fold-change values compared to the GSPE-untreated/non-DENV-infected Huh-7 cells. Data are presented as mean ± SD of three independent experiments. ** *p* < 0.01.

The NF-κB and the MAPK signaling pathways are associated with the regulation of COX-2 expression [29,30]. To explore whether GSPE suppresses DENV-mediated COX-2 expression by interfering with NF-κB signaling, we performed an NF-κB-mediated transcription reporter assay. Huh-7 cells were transfected into a pNF-κB-Luc reporter plasmid containing the luciferase reporter gene linked to five repeats of the NF-κB binding sites and then infected with DENV-2. After a 3-day treatment with increasing concentrations of GSPE, GSPE concentration dose-dependently decreased luciferase activity compared to untreated Huh-7 cells (Figure 4a), indicating that the GSPE-mediated NF-κB transcriptional activation suppressed DENV replication. We further confirmed that GSPE interrupted nuclear translocation of p65 NF-κB in a concentration-dependent manner (Figure 4b). We next investigated the effect of GSPE on MAPK signaling molecules, including extracellular regulated protein kinases 1 and 2 (ERK1/2), p38 kinase, and c-Jun NH2-protein kinase (JNK), in relation to suppression of COX-2 expression. To accomplish this, we treated DENV-infected Huh-7 cells with 20 μg/mL of GSPE for 0–120 min and then examined the effect of GSPE on the phosphorylation status of these factors by western blotting. As shown in Figure 4c, GSPE suppressed the proportions of the phospho-ERK and phospho-p38 proteins compared to those of total-ERK and total-p38, respectively, in a time-dependent manner (upper and middle panels). No significant effect was observed on the phospho-JNK protein level in GSPE-treated Huh-7 cells when compared to the total JNK protein level (bottom panels). Taken together, these results reveal that GSPE blocks the NF-κB and ERK/P38 MAPK signaling pathways to attenuate COX-2 expression and suppress DENV replication.

### 3.5. GSPE Prolongs the Life of DENV-Infected ICR Suckling Mice

**Figure 5:**
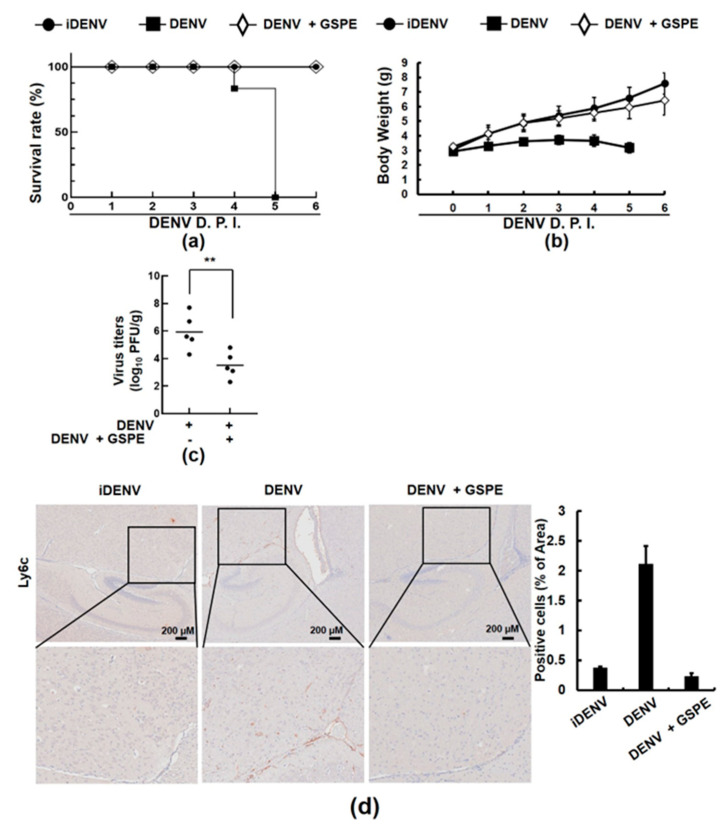
GSPE protects ICR suckling mice from DENV-caused death. Six-day-old ICR suckling mice were intracerebrally injected with 1 × 10^5^ PFU of DENV-2 or iDENV. GSPE (20 mg/kg) was intracerebrally administered at 1, 3, and 5 dpi. The survival rates (**a**) and body weights (**b**) of the mice were measured daily after viral infection and GSPE treatment. (**c**) The DENV titer in the brain was determined by plaque assay. Each point was calculated from an average of all animals in the group (n = 5). ** *p* < 0.01. (**d**) Monocyte infiltration was examined by IHC staining using an anti-Ly6C antibody and further quantified with ImageJ software from at least five random images of each group, each of which contained five mice. The results were collected and plotted daily until the mice died or were sacrificed at day 6 dpi by CO_2_ asphyxiation.

To evaluate the antiviral efficacy of GSPE in vivo, 6-day-old ICR suckling mice were intracerebrally injected with 1 × 10^5^ PFU of DENV-2 and then injected with 20 mg/kg of GSPE 1-, 3-, and 5-days post-infection (dpi). An injection of heat-inactivated DENV (iDENV) served as the negative control. The survival rates and body weights of the DENV-infected mice with or without the treatment were recorded daily for 6 days. The results showed that the survivability of the DENV-infected suckling mice treated with GSPE increased to about 80% compared with the untreated group (Figure 5a). The GSPE treatment also reduced the weight loss caused by DENV infection (Figure 5b). We demonstrated that the GSPE treatment decreased viral propagation in the suckling mouse brain with a two-fold lower log viral titer value being detected in the DENV-infected brains from the 20 mg/kg GSPE group (Figure 5c). Previous studies have demonstrated that DENV causes viral encephalitis and induces monocyte infiltration in vivo following viral infection, which is caused by inflammation of the brain parenchyma [31]. According to IHC staining experiments conducted with the anti-Ly6C antibody, a marker of human monocytes, GSPE significantly decreased monocyte infiltration compared with the GSPE-untreated control group (Figure 5d). Collectively, these results suggest that GSPE is a potential supplementary treatment against DENV and related neurological disorders.

## 4. Discussion

Natural products from medicinal plants or fruits are ideal alternatives to combat DENV because of their virucidal properties. In this study, GSPE effectively suppressed the DENV replication of four DENV serotypes (Figure 1). In addition, DENV infection stimulated an inflammatory response by activating various host cellular factors, including COX-2 and its PGE_2_ metabolite. Modulating COX-2 expression using selective compounds or plant extracts is an excellent strategy to regulate viral replication and viral-induced inflammation [8,32]. Several studies have demonstrated that GSPE alleviates viral or disease-induced inflammation by interrupting COX-2 and its metabolites [24,33]. According to an animal model study, a dietary GSPE supplement protects against intestinal tumorigenesis by reducing COX-2 protein levels by 56–64% in APC (min/+) mice [34]. In the present study, we demonstrated that GSPE suppressed DENV replication by attenuating COX-2 expression (Figure 2 and Figure 3). These findings are similar to those of our previous study in which GSPE significantly suppressed HCV replication by reducing COX-2 expression [24]. Moreover, NF-κB and MAPK are two important factors associated with the COX-2 signaling pathway. A previous study found that GSPE strongly suppressed azoxymethane (AOM)-induced colon tumorigenesis by blocking NF-κB and MAPK signaling molecules [35]. Blocking the NF-κB and MAPK signaling pathways is a potential strategy against DENV replication and pathogenesis [8]. Our results showed that GSPE inhibits DENV replication by blocking the NF-κB and ERK/p38 MAPK signaling pathways (Figure 4). Based on our findings, we propose that GSPE could be administered as a dietary supplement to prevent DENV replication and infection. 

Oxidative stress is another important factor that contributes to the progression of dengue diseases such as dengue hemorrhagic and plasma leakage conditions [36]. Reactive oxygen species production and inflammation following viral infection contribute to the development of neuroinvasive diseases such as the Japanese encephalitis virus, West Nile virus, and DENV [37]. In contrast, antioxidant molecules normalize oxidative stress and reduce the pathogenesis of DENV [38]. Many studies have demonstrated the use of antioxidants to treat viral infections causing encephalitis or subarachnoid hemorrhage [39]. Additionally, many studies have demonstrated that GSPE has strong antioxidant properties [40,41,42]. According to our study, 20 mg/kg of GSPE increased the survival rate of DENV-infected ICR suckling mice while simultaneously lowering the viral load and preventing monocyte infiltration in the brain (Figure 5). These findings suggest that supplementary use of GSPE during a DENV infection could prevent the progression of the infection and its pathogenesis. Further research is required to identify the antioxidant role of GSPE in controlling the progression of DENV infection.

DENV is an infectious trigger for an acute vascular disorder associated with the inflammatory process. DENV infection induces inflammatory cytokines and pro-inflammatory molecules, including IL-1 β, IL-10, IFN-γ, TNF-α, and iNOS, which are associated with DENV pathogenesis [43]. A previous study demonstrated that activating NF-κB by DENV infection is directly linked to the synthesis of iNOS/NO and TNF-α in RAW264.7 cells [44]. Moreover, pro-inflammatory molecules, such as TNF-α and iNOS, facilitate endothelial permeability leading to vascular leakage and hemorrhage [45,46]. In this study, the GSPE treatment substantially alleviated DENV-induced cytokine production, including TNF-α, IL-1, iNOS, IL-6, and IL-8, in DENV-infected Huh-7 cells (Figure S1). Another study showed that quercetin, a bioactive compound in GSPE, has a protective effect on DENV-induced pro-inflammatory cytokines, such as IL-6 and TNF-α, and slight effects on IL-10 and IFN-γ [47]. Based on the inhibitory effect of NF-κB and the anti-inflammatory profile of GSPE, we propose that GSPE improved DENV-induced vascular leakage and hemorrhaging by opposing iNOS/NO and TNF-α synthesis. In this study, we found that GSPE significantly reduced the viral load and monocyte infiltration, which suggests a reason for its potency against the DENV-induced hemorrhagic effect. Additionally, GSPE contains many bioactive constituents which may synthetically inhibit DENV infection and virus-caused diseases. Therefore, further investigation is needed to evaluate the anti-hemorrhagic effect of GSPE in vivo using 6–8-week-old AG129 mice infected with DENV through different administration routes such as intraperitoneal or intravenous injection [48]. Additionally, GSPE contains many bioactive constituents which may synthetically inhibit DENV infection and virus-caused diseases. The multiple potential targets of GSPE against DENV infection are also worthy of further investigation.

**Figure 6:**
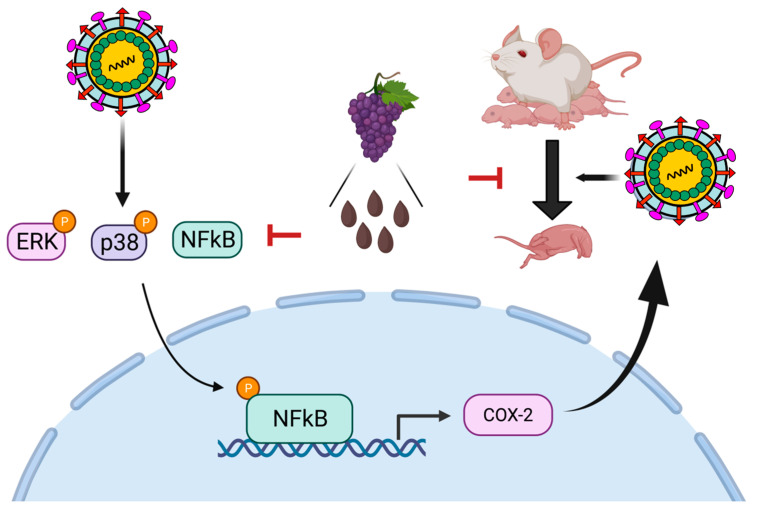
Proposed model illustrating the inhibitory mechanism of GSPE on DENV replication and the DENV-induced inflammatory response. GSPE suppresses DENV replication by downregulating COX-2 expression and reducing activity in the NF-κB and MAPK/ERK/P38 signaling pathways, ultimately reducing the threat of death from dengue infection in DENV-infected mice.

Plant extracts have been widely applied as medicines since ancient times due to their multi-functional properties, accessibility, cost-effectiveness, and good safety profiles [49,50]. Similarly, GSPE is frequently used as a dietary supplement because of its broad-spectrum therapeutic benefits, including antioxidant, antimicrobial, anticarcinogenic, and anti-inflammatory effects, which are due to the presence of high amounts of polyphenolic compounds (60–70%) [41]. Many studies have discussed that resveratrol and quercetin are two promising polyphenols contained in GSPE that exert anti-DENV activity. Treatment with 50 μg/mL quercetin lowered DENV-2 RNA levels by 67% in a cell-based assay [9]. Molecular docking and molecular dynamics studies have shown that DENV NS5 polymerase or NS3 protease are targets of quercetin [51,52]. Furthermore, resveratrol blocks the translocation of high mobility group box 1, ultimately leading to the production of interferon-stimulated genes against DENV infection [53]. Respective treatments with resveratrol derivatives, including 250 nM PNR-4-44 and PNR-5-02, inhibit DENV-2 by 89% and 91, respectively [54]. Moreover, catechin, epicatechin, and procyanidins are the most frequent phenolics in GSPE [26]. A previous study demonstrated that catechin extracted from *Psidium guajava* inhibits DENV-2 replication (>90%) in in vitro and in silico studies [55]. Although several studies have shown that phenolic compounds in GSPE possess anti-DENV activity, the molecular mechanisms of how the GSPE bioactive compounds assert their anti-DENV activity have not been identified. In the present study, we demonstrated that GSPE inhibited DENV replication by modulating COX-2 expression and inactivating the NF-κB and ERK/p38 MAPK signaling pathways (Figure 6). Collectively, the availability of various anti-DENV compounds in GSPE suggests that supplementary use of GSPE is a new, effective, and alternative therapeutic option to treat DENV infection because of the synergistic effect of multiple active substances with different targets against DENV replication.

## Figures and Tables

**Table 1:** Oligonucleotide sequences for real-time RT-PCR.

Oligonucleotide Name	Sequence 5′-3′
5′NS5	5′-GGA AAC CAA GCT GCC CAT CA-3′
3′NS5	5′-CCT CCA CGG ATA GAA GTT TA-3′
5′TNF-α	5′-CCT GTG AGG AGG ACG AAC-3′
3′TNF-α	5′-AAG TGG TGG TCT TGT TGC-3′
5′IL-1β	5′-GGA GAA TGA CCT GAG CAC-3′
3′IL-1β	5′-GAC CAG ACA TCA CCA AGC-3′
5′iNOS	5′-CTT TGG TGC TGT ATT TCC-3′
3′iNOS	5′-TGT GAC CTC AGA TAA TGC-3′
5′COX-2	5′-CCG AGG TGT ATG TAT GAG-3′
3′COX-2	5′-TGG GTA AGT ATG TAG TGC-3′
5′GAPDH	5′-GTC TTC ACC ACC ATG GAG AA-3′
3′GAPDH	5′-ATG GCA TGG ACT GTG GTC AT-3′

## Data Availability

The data presented in this study are available on request from the corresponding author.

## Supplementary Materials

The following supporting information can be downloaded at: https://www.mdpi.com/article/10.3390/v15040884/s1, Figure S1: GSPE inhibits DENV-induced pro-inflammatory cytokine gene expression.
