# Supplementary material for: Grape Seed Proanthocyanidins Inhibit Replication of the Dengue Virus by Targeting NF-kB and MAPK-Mediated Cyclooxygenase-2 Expression
Source: Viruses. 2023 Mar 30;15(4):884. doi: 10.3390/v15040884 (PMC10140912; doi:10.3390/v15040884)
Supplement: Supplementary file 1 [file viruses-15-00884-s001.zip › viruses-2224354-supplementary.pdf]

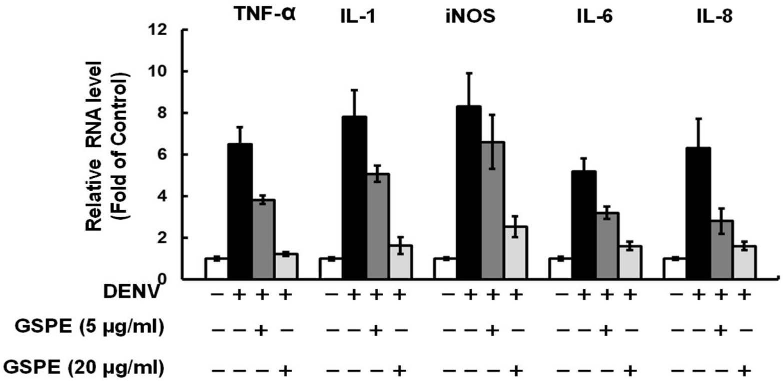

Supplementary Figure S1. GSPE inhibits DENV-induced pro-inflammatory cytokine gene expression. Huh-7 cells were infected with DENV-2 at an MOI of 0.1 for 2 h. The cells were incubated with 5 or 20 μg/mL of GSPE. Total RNA was extracted 3 days later. Gene expression of inflammatory cytokines, including TNF-*α*, IL-1, iNOS, IL-6, and, IL-8, was quantified by RT-qPCR following normalization of cellular *gapdh* mRNA. The relative expression levels of the inflammatory cytokine genes in non-infected/non-transfected Huh-7 cells were defined as 1. Data are presented as mean ± SD of three independent experiments.
